# Ferroptosis regulation in doxorubicin-induced cardiotoxicity: multi-mechanism interventions and translational strategies

**DOI:** 10.3389/fphar.2026.1852342

**Published:** 2026-07-02

**Authors:** XiaoJie Chen, YuYuan Lu, YiYuan Wang, YuShun Kou, YuanHui Gu, Lin Yi

**Affiliations:** 1 School of Traditional Chinese and Western Medicine, Gansu University of Chinese Medicine, Lanzhou, Gansu, China; 2 Laboratory of Traditional Chinese and Western Medicine, Gansu Provincial Hospital, Lanzhou, Gansu, China; 3 Department of General Surgery, Gansu Provincial Hospital, Lanzhou, Gansu, China; 4 Chronic Disease Laboratory, Gansu University of Chinese Medicine, Lanzhou, Gansu, China

**Keywords:** doxorubicin-induced cardiotoxicity, ferroptosis, gut microbiota-heart axis, phytochemicals, tissue-targeted intervention

## Abstract

Doxorubicin (DOX), a highly effective anthracycline chemotherapy drug, has its clinical application severely restricted by dose-dependent doxorubicin-induced cardiotoxicity (DIC). Ferroptosis, an iron-dependent, lipid peroxidation-driven regulated cell death, has been confirmed as a core pathological mechanism in DIC, where it synergistically participates with multiple cell death modalities in myocardial injury. This review systematically elaborates the multidimensional molecular mechanisms underlying DOX-induced myocardial ferroptosis, including: lipid peroxidation cascade self-amplification, bidirectional regulation by selective autophagy, multilayered GPX4 modification and stability regulation, and involvement of the gut microbiota-heart axis. Concurrently, we summarize evidence for natural phytochemicals—including flavonoids, polyphenols, terpenoids, and traditional Chinese medicine compounds—that inhibit myocardial ferroptosis via multi-target mechanisms, providing theoretical support for phytochemical-based DIC intervention. Critically, this review addresses the clinical translation dilemmas in ferroptosis regulation and proposes three innovative strategies: (1) time-decoupling strategy based on IFN-γ signaling; (2) cardiac-targeted nanodrug delivery system employing the OGF/OGFR axis; (3) cross-regulation between ferroptosis and other regulated cell death pathways. These strategies aim to achieve tissue-selective ferroptosis intervention, simultaneously protecting myocardium while maintaining DOX anti-tumor efficacy, thereby providing molecular mechanistic basis and clinical translational directions for constructing precision ferroptosis-targeted cardioprotective strategies.

## 1 Introduction

Doxorubicin (DOX) is one of the most effective anthracycline chemotherapeutic agents in clinical oncology. However, its clinical application is significantly limited by dose - dependent doxorubicin - induced cardiotoxicity (DIC) (Lyon et al., 2022; Minotti et al., 2004). The hallmark of DIC is its insidious progression: at the sub-cellular level, cardiac dysfunction develops progressively but often without obvious early clinical symptoms. If not intervened in a timely manner, the condition can progress to congestive heart failure, ultimately leading to irreversible myocardial damage (Armenian et al., 2017; Cardinale et al., 2010; Lyon et al., 2022; Takemura and Fujiwara, 2007). This insidious progression often causes delays in diagnosis and treatment, thereby exacerbating the irreversibility of myocardial damage (Cardinale et al., 2010; Lyon et al., 2022).

The molecular pathogenesis of DIC is highly complex, and its core mechanism involves the interaction between the cellular stress response and the programmed cell death pathway. Doxorubicin exerts its cardiotoxic effect mainly by specifically inhibiting topoisomerase IIβ (Top2β), which in turn induces DNA damage. This initial damage triggers a series of downstream cascade reactions, including mitochondrial dysfunction, excessive production of reactive oxygen species (ROS), and iron metabolism disorders. These abnormalities jointly promote lipid peroxidation (Minotti et al., 2004; Takemura and Fujiwara, 2007; Zhang et al., 2012). The study by Zhang et al. (2012) further confirmed that Top2β - mediated DNA damage is the initiating event of this pathological cascade reaction. Various forms of cell death such as apoptosis, necroptosis, and ferroptosis (and potentially pyroptosis and cuproptosis), also disrupt the normal autophagic flux within cardiomyocytes (Yang F. et al., 2024). It is the synergistic effect of these pathological processes that leads to the continuous loss of cardiomyocytes, persistent inflammatory infiltration, pathological myocardial fibrosis, and associated adverse ventricular remodeling. Eventually, these cumulative changes gradually induce heart failure and irreversible cardiomyopathy (Minotti et al., 2004; Yang F. et al., 2024).

The cumulative dose of doxorubicin is the main determinant of DIC risk, but there are significant individual differences in susceptibility, which is particularly prominent in children and adolescents. Their high susceptibility may be related to the fact that cardiomyocytes in this age group are still in the developmental stage, making them more sensitive to the toxic effects of doxorubicin. The review by Minotti et al. (2004) indirectly supports this view.

This difference is closely related to the characteristics of myocardial development. Factors such as myocardial metabolic capacity, endogenous antioxidant capacity, mitochondrial maturity, and the integrity of the DNA damage repair mechanism change with the developmental stage, which in turn affects individual sensitivity to doxorubicin - induced cardiotoxicity (Armenian et al., 2015; Cardinale et al., 2010; Lipshultz et al., 2013; Lyon et al., 2022; Minotti et al., 2004).

The current core strategies for the clinical prevention and treatment of DIC mainly include three aspects: controlling the dosage, conducting systematic cardiac monitoring, and implementing cardioprotective intervention measures for high - risk groups (Armenian et al., 2017; Lyon et al., 2022). Currently, dexrazoxane is the only cardioprotective drug approved by the US FDA, but its clinical application still has obvious limitations. The main reason is that this drug has the risk of reducing the anti - tumor efficacy and increasing the risk of treatment - related secondary malignancies (De Baat et al., 2022; Lyon et al., 2022).

Therefore, the development of a new generation of cardioprotective drugs has become a research hotspot. Among them, ferroptosis, as a key regulatory node in DIC - induced myocardial injury, has shown good therapeutic potential with its inhibitors (Fang et al., 2019; Wu et al., 2024). Meanwhile, the cardioprotective value of natural bioactive compounds has also gradually attracted attention. These phytochemicals and dietary components can play a protective role through multiple pathways, such as reducing oxidative stress, maintaining the stability of mitochondrial structure and function, and regulating the cell death pathway, providing a theoretical basis for the development of new cardioprotective strategies (Yi et al., 2024).

## 2 Clinical phenotypes and molecular mechanisms of DOX-induced cardiotoxicity

DIC primarily presents two clinical phenotypes: acute and chronic (Armenian et al., 2017; Feher et al., 2020; Kong et al., 2022; Lyon et al., 2022; Planek et al., 2020; Rawat et al., 2021). Acute toxicity is mainly characterized by abnormal electrocardiograms and arrhythmias. In some cases, transient cardiac dysfunction may occur. The progression of chronic toxicity is characterized by insidiousness, lacking obvious early warning signals. It gradually impairs myocardial function, ultimately leading to persistent left ventricular systolic dysfunction and adverse cardiac remodeling, and can progress to heart failure. The research evidence of Kong et al. (2022) confirms its characteristics of subtle and difficult - to - detect early manifestations. Combining current clinical guidelines and related research (Armenian et al., 2017; Feher et al., 2020; Kong et al., 2022; Lyon et al., 2022; Planek et al., 2020; Rawat et al., 2021), early detection and timely intervention are crucial steps to improve the prognosis of such patients and reduce the incidence of long - term adverse cardiac events, which are of indispensable clinical significance.

From the perspective of tissue and cell levels, the histological characteristics of DIC are clear, mainly manifested by disordered arrangement and atrophy of cardiomyocytes, accompanied by interstitial fibrosis changes (Birdal et al., 2024; Fernandez-Chas et al., 2018; Song et al., 2024). At the biochemical level, this pathological process is accompanied by significant inflammation activation, which can be specifically reflected in the elevation of inflammation - related indicators such as lactate dehydrogenase, creatine kinase, interleukin - 6, and tumor necrosis factor - α. Meanwhile, there is an obvious oxidative stress reaction, which can be confirmed by the increase of malondialdehyde, a lipid peroxidation product, and the depletion of endogenous antioxidant substances such as glutathione and catalase (Birdal et al., 2024; Song et al., 2024; Songbo et al., 2019). The study by Song et al. (2024) further verifies the synergistic effect of inflammation and oxidative stress in DIC - induced myocardial damage.

Molecular - level research shows that myocardial topoisomerase IIβ (Top2β) is the core mediator of doxorubicin (DOX) - induced cardiotoxicity. DOX binding to Top2β stabilizes the TOP2β-DNA complex, converting this normally transient intermediate into a persistent cleavage complex that causes double-strand DNA breaks, leading to abnormal gene transcription, impaired mitochondrial function, and the large - scale production of reactive oxygen species (ROS) (Cui et al., 2019; Kong et al., 2022; Zhang et al., 2012). These damage factors interact with each other, jointly leading to abnormal mitochondrial structure and function, impaired mitochondrial autophagy, decreased membrane potential, and ATP depletion, ultimately accelerating the failure of myocardial energy metabolism (Kong et al., 2022; Wu B. B. et al., 2022). Wu B. B. et al. (2022) also clarified the core role of mitochondrial dysfunction in the progression of DIC in their study.

In addition to the above - mentioned widely confirmed classical mechanisms, the latest research indicates that cuproptosis, as a new type of programmed cell death mode, is also involved in mediating the pathological damage process of DIC (Chen et al., 2025). To clarify the regulatory role of cuproptosis in DIC and promote the clinical translation of relevant results, comprehensive human trials are urgently needed for verification. At present, the verification work can be completed through various means: directly detecting the copper ion content in peripheral blood and myocardial tissue (Yang et al., 2023); analyzing the expression characteristics of cuproptosis - related genes, including the FDX1 gene and genes encoding lipoylated tricarboxylic acid cycle enzymes (Cobine and Brady, 2022; Li D. et al., 2022; Yang et al., 2023); screening specific circulating biomarkers, with a focus on exploring potential autoantibodies (such as anti - FDX1 antibodies).

## 3 Molecular regulation of ferroptosis and its dual roles in cardio-oncology

Ferroptosis was officially defined by Dixon in a study in 2012 (Dixon et al., 2012). As a unique form of iron - dependent regulated cell death, its most prominent feature is the uncontrolled process of lipid peroxidation (Alves et al., 2025; Stockwell, 2022). Different from apoptosis, its occurrence does not require the participation of caspases and apoptosis - related molecular mechanisms. Combining with Stockwell’s decade - long summary research in the field of ferroptosis (Stockwell, 2022), it is confirmed that the core difference between ferroptosis and traditional cell death lies in the unique mechanism driven by iron dependence and lipid peroxidation.

The core molecule for the execution of ferroptosis is glutathione peroxidase 4 (GPX4). This enzyme uses reduced glutathione (GSH) as a substrate to reduce phospholipid hydroperoxides on the cell membrane to inactive lipid alcohols, thereby effectively inhibiting lipid peroxidation and maintaining the intracellular redox homeostasis (Alves et al., 2025; Liang et al., 2022; Stockwell, 2022). Once the activity of GPX4 is inhibited, or GSH is depleted by blocking the Xc^−^ transport system (encoded by SLC7A11 gene), the cascade reaction of ferroptosis will be rapidly initiated. Acyl - CoA synthetase long - chain family member 4 (ACSL4) is another key regulatory factor, whose function is to integrate polyunsaturated fatty acids (PUFA) into cell membrane phospholipids. Studies have shown (Gan, 2022; Lei et al., 2021; Liang et al., 2022) that this substrate modification significantly enhances the cell’s sensitivity to ferroptosis, especially in the scenario of ionizing radiation exposure, where this regulatory effect is more prominent.

In the oxidative stress microenvironment induced by doxorubicin (DOX), the cell’s sensitivity to ferroptosis is not regulated by a single factor but depends on the synergistic action of iron homeostasis, lipid homeostasis, antioxidant defense system, and selective autophagy (Abe et al., 2022; Fang et al., 2019; Wang et al., 2025; Ye H. et al., 2024). The KEAP1 - NRF2 signaling axis is a typical representative in this regulatory network. After NRF2 is transcriptionally activated, it specifically up - regulates ferroptosis - protective genes such as SLC7A11 and GPX4, ultimately endowing cells with ferroptosis resistance (Lei et al., 2021; Sun et al., 2023). It is worth noting that the regulation of ferroptosis by autophagy is bidirectional. On the one hand, selective autophagy can play an anti - ferroptosis role by removing abnormal lipids and maintaining redox balance. On the other hand, it can promote the degradation of GPX4 through an acid sphingomyelinase (ASM) - dependent pathway, thereby promoting the occurrence of ferroptosis (Thayyullathil et al., 2021; Wang et al., 2025; Ye L. et al., 2024). This dual - regulatory mode also provides important targets for subsequent intervention strategies.

Ferroptosis has been proven to be a therapeutic target with extremely high clinical application value, especially in the field of cardio - oncology, where its dual therapeutic potential is particularly prominent. Inhibiting ferroptosis through specific inhibitors such as ferrostatin - 1 and liproxstatin - 1, or iron chelators can effectively protect cardiomyocytes and reduce chemotherapy - induced cardiotoxicity (Alves et al., 2025; Dixon et al., 2012; Liang et al., 2022; Stockwell, 2022; Sun et al., 2023). In addition, studies by Hu et al. (2023) and Wen J. et al. (2024) have shown that natural compounds such as tanshinone IIA can also play a cardioprotective role by inhibiting ferroptosis. In contrast, inducing ferroptosis can significantly enhance the anti - tumor effect. A study by Lei et al. (2021) confirmed that when ferroptosis induction is combined with radiotherapy, the anti - tumor efficacy is further improved. This duality in treatment determines that the regulation of ferroptosis needs to be combined with specific tissue scenarios. In myocardial tissue, ferroptosis needs to be inhibited to reduce DOX - induced damage, while in malignant tissue, ferroptosis needs to be promoted to efficiently eliminate cancer cells. Therefore, in - depth analysis of the regulatory mechanism of ferroptosis in cardiomyocytes under the action of DOX and clarification of the differences between protective and damaging pathways are key prerequisites for the rational design of myocardial - selective therapeutic intervention strategies.

Although the execution mechanism of ferroptosis has been fully elucidated, iron homeostasis dysregulation, a key upstream driving factor, is often overlooked. Imbalance in iron homeostasis is the fundamental cause of doxorubicin (DOX)-induced cardiac ferroptosis. A thorough understanding of this mechanism is crucial for formulating effective cardioprotective strategies.

Iron is an essential element for cellular metabolism. However, excessive iron generates a large amount of reactive oxygen species (ROS) through the Fenton reaction, leading to oxidative damage. Cells maintain iron homeostasis through a multi - level and precise mechanism (Dixon and Olzmann, 2024). Iron is mainly taken up by cells through transferrin - TfR1 (transferrin receptor 1) receptor - mediated endocytosis and direct uptake by DMT1 (divalent metal transporter 1) (Liu C. et al., 2022). Once inside the cell, iron either enters the labile iron pool (LIP) for metabolic processes such as mitochondrial respiration and DNA synthesis, or is stored by ferritin (FTH1/FTL), or is exported the only cellular iron pump, ferroportin (FPN1) (Chen et al., 2024a). This system is controlled by two major regulatory axes: at the cellular level, iron - regulatory proteins (IRPs) and iron - responsive elements (IREs) form a “molecular switch”. In case of iron deficiency, the IRP/IRE system upregulates iron - uptake proteins such as TfR1 and downregulates iron - storage and - export proteins such as FTH1/FTL/FPN1. The opposite occurs when iron is sufficient (Liu X. et al., 2022). At the systemic level, hepcidin, synthesized by the liver, controls iron release by promoting the degradation of FPN1, thereby regulating systemic iron balance (Zhao Y. et al., 2025).

**FIGURE 1:**
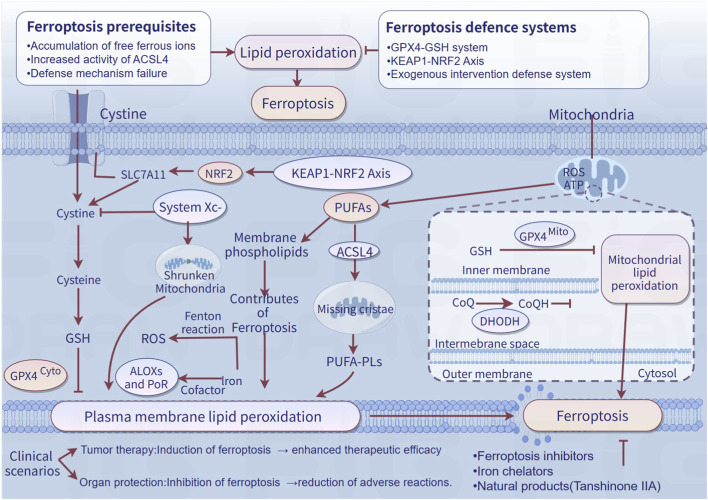
The initiation of ferroptosis primarily stems from the cellular accumulation of excess free ferrous ions (Fe2+), which directly drive the generation of reactive oxygen species (ROS) via the Fenton reaction. Concurrently, Fe2+ serves as a critical cofactor for both lipoxygenases (ALOXs) and NADPH oxidases (PoR), thereby accelerating the lipid peroxidation of membrane phospho-lipids, particularly those rich in polyunsaturated fatty acids (PUFA-PLs). Upon accumulation of lipid peroxidation products to a critical threshold, overwhelming the scavenging capacity of cru-cial defense systems such as glutathione peroxidase 4 (GPX4), significant damage to the plasma membrane ensues, ultimately precipitating cell death, a process termed ferroptosis. Consequently, the dysregulation of iron homeostasis emerges as a pivotal determinant in the onset of ferroptosis.

DOX disrupts this balance through multiple mechanisms: it directly enhances the expression of TfR1 and DMT1, promoting iron uptake. Meanwhile, DOX - induced ROS damage the IRP/IRE feedback system, inhibiting the expression of iron - storage and - export proteins (FTH1/FTL/FPN1) (Liu X. et al., 2022). This “pathological triad” (i.e., increased uptake, decreased storage, and decreased export) results in pathological iron accumulation and abnormal expansion of the LIP. Excessive iron in the LIP catalyzes the Fenton reaction to generate a large amount of ROS, accelerating lipid peroxidation, which is a key upstream event triggering cardiac ferroptosis (Mou et al., 2019; Sun et al., 2023). This imbalance in iron metabolism forms a complex cross - regulatory network with lipid peroxidation, autophagy, GPX4 regulation, and mitochondrial dysfunction (Li J. et al., 2023), further increasing the susceptibility of cardiomyocytes to ferroptosis (Figure 1).

## 4 Multi-dimensional molecular mechanisms of DOX-induced myocardial ferroptosis

### 4.1 The lipid peroxidation cascade and transcriptional dysregulation in DOX-induced ferroptosis

Ferroptosis has been confirmed as the key mechanism of doxorubicin (DOX)-induced myocardial injury, and uncontrolled lipid peroxidation is the core link in this pathological process (Fang et al., 2019; Miotto et al., 2020; Pedrera et al., 2025; Tadokoro et al., 2023; Zilka et al., 2017). In essence, it is a type of self-amplifying free-radical chain reaction: ferrous ions (Fe^2+^) catalyze the decomposition of lipid hydroperoxides (LOOH), initiate and continuously generate lipid free radicals, and ultimately break through the cellular antioxidant defense system, leading to extensive damage to the membrane structure (Li et al., 2020; Miotto et al., 2020; Tadokoro et al., 2023). Cardiomyocytes are particularly sensitive to oxidative stress due to their high mitochondrial density and membranes rich in polyunsaturated phospholipids, which also makes them susceptible target cells for ferroptosis (Liu et al., 2022a). The lipophilic free-radical scavenger ferrostatin-1 (Fer-1) can block the chain reaction by directly neutralizing lipid free radicals, thus effectively inhibiting the occurrence of ferroptosis (Miotto et al., 2020; Zilka et al., 2017).

Lipid peroxidation mediates cell damage mainly through two pathways. On the one hand, the aldehyde products such as malondialdehyde MDA and 4-hydroxynonenal (4-HNE) generated by the peroxidation reaction can covalently modify transmembrane proteins, damage membrane integrity, and interfere with membrane-dependent functions (Li et al., 2020; Liu, et al., 2022a). Under an electron microscope, mitochondrial fragmentation can be observed in the early stage of ferroptosis, and further development to sarcolemma rupture in the late stage (Liu, et al., 2022a; Pedrera et al., 2025; Tadokoro et al., 2023). On the other hand, polyunsaturated fatty acids (PUFAs), especially arachidonic acid, are lipid substrates that are extremely prone to oxidation. DOX can upregulate the expression of ACSL4, promote the incorporation of arachidonic acid into membrane phospholipids, and further amplify the susceptibility to ferroptosis by increasing the level of oxidizable substrates (Cui et al., 2024; Li et al., 2020; Tadokoro et al., 2023).

Existing studies further reveal that lipid peroxidation does not occur randomly but shows obvious subcellular spatial specificity. Its initiation sites are mostly concentrated in the endoplasmic reticulum-mitochondria (ER-MITO) contact area. Such microdomains are rich in oxidizable lipids and iron-metabolism-related proteins, providing an ideal microenvironment for initiating lipid peroxidation (Sassano et al., 2025). Once initial peroxidative damage is formed at the ER-MITO interface, it can rapidly spread along the membrane network, exacerbating the overall damage to myocardial tissue and inducing extensive abnormal cardiomyocyte functions (Christidi and Brunham, 2021; Pedrera et al., 2025).

**FIGURE 2:**
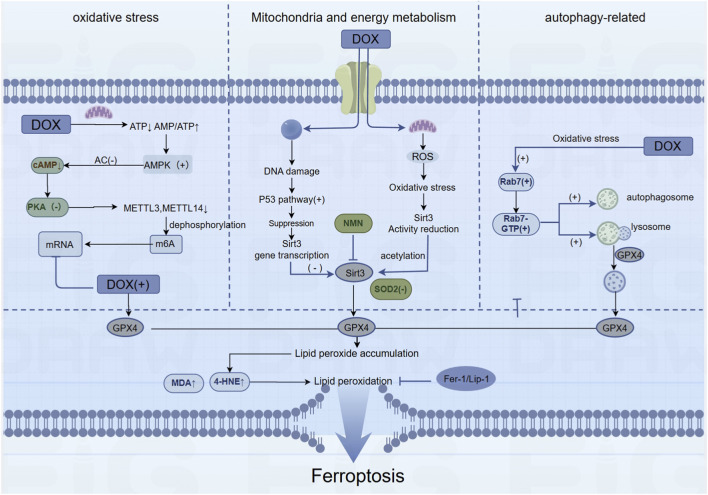
Doxorubicin (DOX) mediates lipid peroxidation and ultimately induces ferroptosis through multiple intricate signaling pathways. Under oxidative stress conditions, DOX exposure leads to a diminished ATP/AMP ratio, which activates AMPK and simultaneously inhibits ade-nylate cyclase (AC), consequently reducing cAMP levels and PKA activity. This cascade is ac-companied by decreased expression of METTL3 and METTL14, thereby altering m6A modification and subsequently influencing GPX4 expression and activity, ultimately potentiating lipid peroxi-dation. Within the mitochondrial and energy metabolism pathway, DOX elicits both DNA damage and reactive oxygen species (ROS) accumulation. DNA damage activates the P53 pathway, leading to suppression of Sirt3 transcription. Concurrently, ROS-induced oxidative stress diminishes Sirt3 activity. While NMN positively modulates Sirt3 expression by mitigating P53-mediated transcrip-tional suppression, reduced SOD2 activity (indicated as SOD2 (−)) further impairs Sirt3 activity via increased acetylation. The overall inhibition of Sirt3 subsequently downregulates GPX4 levels, thereby exacerbating lipid peroxidation. Furthermore, DOX-mediated oxidative stress activates Rab7, which promotes the formation of autophagosomes and lysosomes that subsequently degrade GPX4 through the autophagosome-lysosome pathway. Ultimately, these intricate pathways col-lectively result in the inhibition or degradation of GPX4, culminating in the accumulation of lipid peroxidation products such as malondialdehyde (MDA) and 4-hydroxynonenal (4-HNE), conse-quently triggering ferroptosis. This entire process can be effectively blocked by Fer-1/Lip-1.

Transcriptional-level regulation is also involved in the process of ferroptosis in DOX - induced cardiotoxicity. The NRF2-BACH1 axis is a key regulatory node in this process: NRF2 can activate ferroptosis-protective genes such as SLC7A11, GPX4, and FTH1, while BACH1 competitively binds to inhibit the above-mentioned antioxidant and iron-storage-related programs (Irikura et al., 2023; Nishizawa et al., 2020; Nishizawa et al., 2023; Wang et al., 2024b). DOX intervention can break this balance, inhibit the NRF2 signal, and enhance the activity of BACH1 simultaneously, resulting in a sharp decline in the cellular antioxidant capacity and promoting the progression of ferroptosis (Irikura et al., 2023; Nishizawa et al., 2020; Nishizawa et al., 2023; Wang B. et al., 2023; Wang et al., 2024b; Yin et al., 2025; Yu et al., 2023). This imbalance in transcriptional regulation and the lipid peroxidation cascade reaction synergize with each other, jointly mediating the final execution process of DOX-induced ferroptosis in cardiomyocytes (Figure 2).

### 4.2 Autophagic and mitochondrial regulation of DOX-induced ferroptosis

Doxorubicin (DOX)-induced ferroptosis in cardiomyocytes is modulated by the autophagy network, which exhibits a marked dual regulatory nature: pathological hyperactivation of autophagy accelerates ferroptotic progression, whereas basal autophagic flux exerts cardioprotective effects (He et al., 2024; Hu et al., 2025; Zhou et al., 2022). As a critical regulatory node in cardiomyocytes, the functional outcome of autophagy is not fixed but is instead determined collectively by the activation level and pathway specificity—a characteristic underscoring autophagy’s central role in ferroptosis regulation.

Ferritinophagy, as a core subtype of selective autophagy, is regulated by the autophagy receptor NCOA4. NCOA4 can specifically bind to the ferritin-iron complex, precisely targeting this iron storage form to the autophagosome. After the autophagosome degrades it, free iron ions are released - this process is also a key step in ferritinophagy-mediated iron metabolism (Fang et al., 2021; Zhu et al., 2024).

These released free iron ions will further expand the intracellular redox-active iron pool. The iron ions in this iron pool happen to provide the necessary substrate for the Fenton reaction, which in turn triggers the lipid peroxidation reaction. The continuous accumulation of lipid peroxidation will ultimately accelerate the process of ferroptosis. In the mechanism study of doxorubicin-induced ferroptosis in cardiomyocytes and the special discussion of NCOA4-mediated ferritinophagy (Chen et al., 2024c; Santana-Codina et al., 2021; Zhou et al., 2022), evidence has been provided to support this regulatory pathway.

In DOX-treated cardiomyocytes, hallmark pathological features include significantly elevated NCOA4 expression, an increased LC3-II/LC3-I ratio, and markedly reduced ferritin levels (Santana-Codina et al., 2021; Shao J. et al., 2025; Zhou et al., 2022)—collectively indicating aberrant activation of the ferritinophagy flux. As demonstrated experimentally by Zhou et al. (2022), this NCOA4-mediated ferritinophagy axis serves as a key upstream triggering mechanism that converts DOX-induced oxidative stress signals into iron-dependent ferroptosis (Santana-Codina et al., 2021; Shao J. et al., 2025).

Beyond ferritinophagy, chaperone-mediated autophagy (CMA) also contributes to the degradation of ferroptosis-related effector molecules, thereby complementing ferritinophagy. Studies indicate that mitochondrial reactive oxygen species (mtROS) can activate the CMA regulatory pathway, leading to the selective proteasomal and lysosomal degradation of GPX4—a critical defense molecule against ferroptosis—thereby directly impairing cardiomyocyte resistance to ferroptotic death (Hirata et al., 2024). Although the mechanism of the mtROS–CMA–GPX4 axis has been clearly elucidated in non-cardiac cell models, its specific role in DOX-induced cardiomyocyte ferroptosis remains unclear; the authors emphasize the need for direct investigations in cardiomyocyte-specific models to delineate its regulatory significance (Hirata et al., 2024; Xie et al., 2023).

In coordination with the above selective autophagy pathways, mitochondria–ER contact sites (MERCs) maintain overall autophagic flux homeostasis under DOX-induced cardiotoxicity. FUNDC1, a key protein located in the mitochondrial outer membrane, plays a central role in stabilizing MERCs and promoting autophagosome biogenesis (He et al., 2024). DOX exposure significantly suppresses FUNDC1 expression, leading to structural disruption of MERCs, which subsequently impairs both the initiation and progression of autophagy, ultimately exacerbating cardiomyocyte injury (He et al., 2024). The research by He et al. (2024) has confirmed that specifically overexpressing FUNDC1 in cardiomyocytes can not only effectively restore the structural integrity of mitochondrial - endoplasmic reticulum coupling (MERC) but also reverse the impaired autophagy function. More importantly, this operation can significantly alleviate the oxidative stress response of cardiomyocytes after doxorubicin (DOX) treatment and reduce ferroptotic cell damage.

**FIGURE 3:**
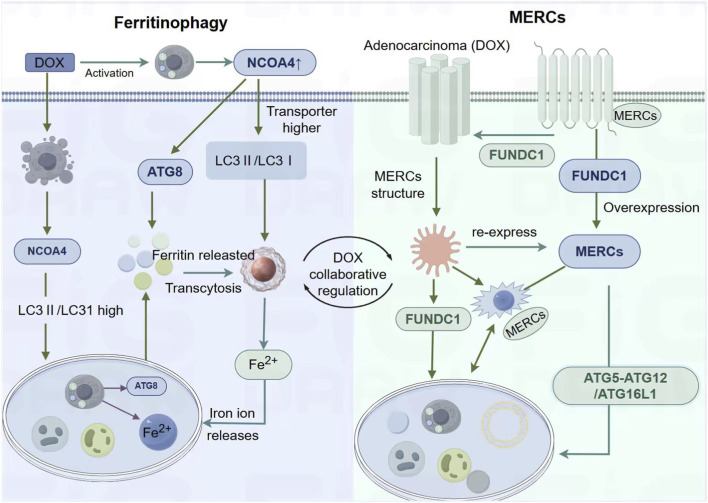
Figure elucidates the intricate mechanisms by which doxorubicin (DOX) orchestrates ferritinophagy and modulates mitochondria-endoplasmic reticulum contact sites (MERCs) in the context of cellular stress responses. DOX drives ferritinophagy—a core catabolic process—by ac-tivating NCOA4, which in turn upregulates autophagy-related proteins LC3 II/LC3 I, culminating in ferritin degradation and subsequent Fe2+ release. Simultaneously, DOX exerts an influence on MERCs architecture within cardiomyocyte cells. Notably, the overexpression of FUNDC1 fur-ther enhances MERCs remodeling and facilitates its engagement with key autophagic machinery, including ATG5-ATG12/ATG16L1. These interconnected events are subject to the synergistic regulation of DOX, underscoring the critical contributions of iron metabolism and organelle crosstalk to DOX-mediated cellular fate decisions.

Combining these experimental results, it is not difficult to find that MERC, autophagy, and ferroptosis do not exist in isolation but form an integrally - related regulatory network. Among them, the structural stability and normal function of mitochondrial contact sites are the core mechanisms determining whether cardiomyocytes are prone to ferroptosis (Li et al., 2024; Ou et al., 2024) (Figure 3).

### 4.3 Multilayered regulation and targeting of GPX4 in DOX-induced cardiotoxicity

Glutathione peroxidase 4 (GPX4), as a core selenoprotein, serves as a crucial defense line for cells against ferroptosis. Its core function lies in reducing phospholipid hydroperoxides (PLOOH) in the lipid bilayer to inert lipid alcohols, thereby maintaining cellular redox homeostasis (Ursini and Maiorino, 2020; Yang et al., 2014). Existing research has clearly indicated that the deficiency or abnormal function of GPX4 can directly lead to the massive accumulation of lipid peroxides, ultimately initiating the ferroptosis program (Xie et al., 2023; Yang et al., 2014). In the doxorubicin (DOX)-induced myocardial injury model, it can be observed that DOX significantly downregulates the expression level and catalytic activity of GPX4 in cardiomyocytes, resulting in the abnormal elevation of lipid peroxidation products such as malondialdehyde (MDA) and 4-hydroxynonenal (4-HNE), and ultimately inducing ferroptosis-dependent myocardial injury. Therefore, GPX4 is a key molecule at the intersection of multiple signaling pathways.

The protein homeostasis of GPX4 is mainly maintained by two post-translational degradation pathways. In cardiomyocytes, the ubiquitin-proteasome system (UPS)-mediated ubiquitination modification is the dominant pathway for regulating GPX4 degradation. The deubiquitinases USP51 and USP10 can reverse the ubiquitination process of GPX4 by cleaving ubiquitin chains, enhancing protein stability and thereby increasing the cell’s tolerance to ferroptosis (Lian et al., 2024; Peng et al., 2024). In contrast, the E3 ubiquitin ligase MG53 catalyzes polyubiquitination of GPX4, accelerating its recognition and degradation by the proteasome and significantly increasing the susceptibility of DOX-treated cardiomyocytes to ferroptosis (Jiang et al., 2024).

In addition to the ubiquitin-proteasome system, chaperone-mediated autophagy (CMA) activated by mitochondrial reactive oxygen species (mtROS) constitutes a secondary pathway for GPX4 degradation [64]. Research suggests that DOX stimulation promotes the massive generation of mtROS in cardiomyocytes and activates CMA, which can selectively recognize GPX4 and mediate its lysosomal degradation. This process can occur independently of ubiquitin signals (Zhao et al., 2024). To achieve the stability of GPX4 protein, it is necessary to simultaneously block the degradation processes mediated by UPS and CMA.

The expression of GPX4 is also regulated at multiple levels, including transcription, post-transcription, and translation. Protein kinase A (PKA) can indirectly regulate the stability of GPX4 transcripts by phosphorylating the RNA m^6^A demethylase ALKBH5, thereby altering the cell’s sensitivity to ferroptosis (Zhao X. et al., 2025). Inhibition of the PKA signaling pathway may reduce the stability of GPX4 mRNA, but this hypothesis still needs further verification in cardiomyocyte models. In addition, m^6^A reader proteins can enhance the binding efficiency of GPX4 mRNA to the translation complex, finely regulating its expression at the post-transcriptional level (Jiang et al., 2021).

At the level of translation and synthesis, the bioavailability of selenium is the rate-limiting step for the production of functional GPX4. Insufficient selenium supply can directly block the translation process of selenoproteins, leading to a decrease in GPX4 protein content (Li Z. et al., 2022). Meanwhile, the mitochondrial NAD^+^-dependent deacetylase SIRT3 can simultaneously regulate GPX4 stability and mitochondrial antioxidant capacity. Downregulation of SIRT3 expression exacerbates lipid peroxidation and increases the risk of ferroptosis (Guo et al., 2024; Liu X. et al., 2022), but its specific molecular mechanism in DOX-induced cardiomyopathy remains to be further elucidated.

**FIGURE 4:**
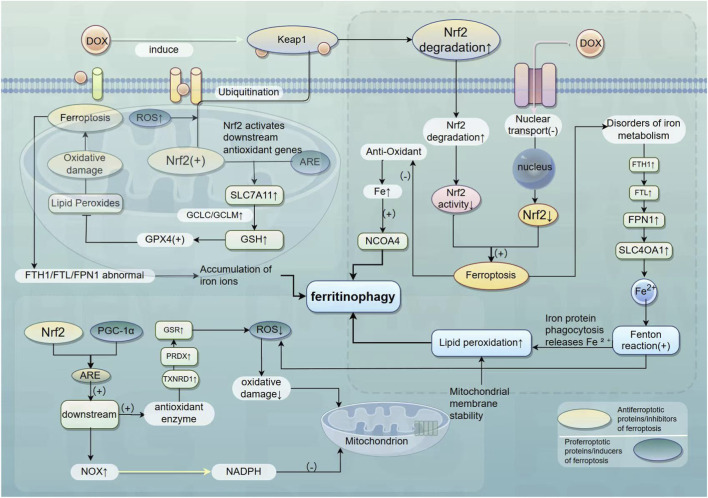
Doxorubicin (DOX) initiates Nrf2 ubiquitination and subsequent degradation via Keap1, resulting in diminished nuclear Nrf2 activity and consequently attenuating the expression of downstream antioxidant genes. This functional impairment of Nrf2 critically disrupts iron meta-bolic homeostasis, manifesting as aberrant levels of FTH1, FTL, and FPN1, which collectively contribute to the intracellular accumulation of iron ions. The accumulating iron ions not only stimulate ferritinophagy but also profoundly exacerbate lipid peroxidation through the Fenton reaction, thereby compromising mitochondrial membrane stability and ultimately precipitating ferroptosis. Although the Nrf2/PGC-1α axis typically orchestrates the defense against reactive oxygen species (ROS) and oxidative damage via antioxidant enzymes, DOX intervention subverts this crucial protective mechanism, consequently driving cells towards ferroptosis.

From the perspective of translational medicine, GPX4 has unique dual-targeting value: inhibiting GPX4 in tumor cells can enhance the ferroptosis-mediated killing effect and improve the anti-tumor treatment outcome; while maintaining the intact function of GPX4 in cardiomyocytes is the core protective strategy against DOX-induced ferroptosis (Wang C. et al., 2023). Targeted protective interventions for the relevant mechanisms of GPX4 can be carried out in three directions: first, maintaining the normal expression of GPX4 by regulating the PKA–ALKBH5–m^6^A axis and ensuring sufficient selenium supply; second, stabilizing the GPX4 protein level by activating deubiquitinases such as USP51/USP10 or inhibiting the activity of E3 ubiquitin ligases; third, providing substrate support for the catalytic function of GPX4 by supplementing glutathione (GSH) and directly scavenging lipid peroxides (Jiang et al., 2024; Lian et al., 2024; Peng et al., 2024). Existing experiments have confirmed that the ferroptosis inhibitors ferrostatin-1 and liproxstatin-1 can directly block the lipid peroxidation chain reaction and maintain the integrity of the cell membrane (Fan et al., 2021). Natural products such as tanshinone IIA and fucoidan can target the NRF2–GPX4 pathway and upregulate the endogenous antioxidant system to exert myocardial protection (Cantrell et al., 2024; Hu et al., 2023; Planek et al., 2020; Wang et al., 2024a; Wen Z. et al., 2024; Wu et al., 2026). At the same time, iron chelators can reduce the free iron pool required for the Fenton reaction and synergistically inhibit ferroptosis with the above intervention measures (Li et al., 2024). Comprehensively targeting the expression, stability, and function of GPX4 and jointly regulating the upstream key molecules of ferroptosis are expected to provide new and feasible translational ideas for the clinical prevention and treatment of DOX-induced myocardial injury (Figure 4).

### 4.4 Emerging pathological mediators: the gut microbiota-heart axis in DOX-induced cardiotoxicity

Beyond the direct molecular mechanisms, emerging evidence reveals that DOX-induced ferroptosis is also regulated by systemic factors, particularly the gut microbiota-heart axis, which also provides a reasonable explanation for the individual differences in patients’ clinical manifestations (Huang et al., 2022; Huang et al., 2025). DOX exposure directly triggers gut microbiota dysbiosis, manifested as a decrease in microbiota diversity, disordered composition structure, and impaired metabolic function (Huang et al., 2022; Huang et al., 2025). The most intuitive feature is the reduction in the levels of beneficial metabolites such as short-chain fatty acids (SCFAs) and secondary bile acids, along with the abnormal accumulation of pathogenic metabolites such as trimethylamine N-oxide (TMAO), along with the abnormal accumulation of pathogenic metabolites in the body (Huang et al., 2025).

Under physiological conditions, the core function of metabolites produced by gut microorganisms is to maintain cardiac energy homeostasis. They ensure energy supply for cardiomyocytes by inhibiting the activity of histone deacetylase (HDAC) and activating the G - protein - coupled receptor (GPR) signaling pathway. Once the microbiota is dysregulated, the metabolic synergy between the host and the microbiota is disrupted, which not only exacerbates the imbalance of cardiac energy metabolism but also reduces the cardiomyocytes’ resistance to oxidative stress (Huang et al., 2024; Kunika et al., 2023).

The mechanisms by which microbiota dysbiosis induces myocardial ferroptosis mainly focus on two core pathways. Firstly, abnormal microbial iron metabolism leads to a significant decrease in the production of butyrate, a key SCFA. This change directly disrupts the balance of intestinal iron ions, ultimately resulting in iron overload in cardiomyocytes (Huang et al., 2022). Excess iron ions catalyze the Fenton reaction, accelerating the process of lipid peroxidation and significantly increasing the sensitivity of cardiomyocytes to ferroptosis. Secondly, microbiota dysbiosis damages the integrity of the intestinal mucosal barrier, the so - called “leaky gut” phenomenon, which leads to the massive release of pro - inflammatory factors such as lipopolysaccharide (LPS) and tumor necrosis factor - α (TNF - α) (Huang et al., 2022; Kunika et al., 2023). Meanwhile, the lack of butyrate impairs the GPR43 signaling pathway required for the differentiation of regulatory T cells (Treg) (Huang et al., 2024). The combination of these factors and the oxidative stress induced by DOX itself leads to unstable activity of glutathione peroxidase 4 (GPX4) and decreased bioavailability of glutathione (GSH), ultimately accelerating the occurrence of myocardial ferroptosis (Kunika et al., 2023).

As an upstream link in the pathogenesis of DIC, the gut microbiota - heart axis provides a new entry point for preventive intervention of the disease. By regulating the gut microbiota, early protection against cardiac injury can be achieved (Huang et al., 2024). Based on existing research results (Huang et al., 2024), prebiotics and dietary fiber can promote the proliferation of SCFA - producing strains and help restore the normal number of Treg cells. Probiotic strains such as Akkermansia muciniphila can directly improve the production efficiency of SCFAs (Huang et al., 2024; Huang et al., 2022; Huang et al., 2025; Kunika et al., 2023). Fecal microbiota transplantation (FMT) can effectively reconstruct the healthy gut microbiota structure (Huang et al., 2024), and fucoidan can play an indirect cardioprotective role by inhibiting the growth of pathogenic bacteria and enriching beneficial strains (Kunika et al., 2023). The common effect of these intervention methods is to reduce DOX - induced myocardial injury by restoring the metabolic synergy between the host and the microbiota, inhibiting lipid peroxidation and the ferroptosis - promoting inflammatory pathway (Huang et al., 2024).

Although therapies targeting the gut microbiota hold great promise, their clinical translation still faces multiple challenges. Firstly, the temporal relationship between gut microbiota dysbiosis and ferroptosis - dependent cardiac injury remains unclear. Determining the dynamic change patterns of the two and finding the optimal early intervention window are still key issues to be addressed in current research (Huang et al., 2024; Huang et al., 2025). Secondly, there are differences in the baseline microbiota composition and eating habits among individuals, and genetic polymorphisms such as the FXR gene also affect treatment outcomes. This requires subsequent research to develop individualized intervention strategies (Huang et al., 2024; Huang et al., 2022; Huang et al., 2025; Kunika et al., 2023). Fecal microbiota transplantation (FMT) and targeted probiotic intervention, as gut microbiota reconstruction strategies, offer a new dimension for the prevention and treatment of doxorubicin-induced cardiotoxicity. FMT restores butyrate-producing bacteria, activates the HDAC inhibition and GPR43 signaling pathways, stabilizes GPX4 activity, and protects GSH bioavailability. Meanwhile, it alleviates ferroptosis sensitivity through Nrf2-mediated mitochondrial quality control (Zhou et al., 2024; Huang et al., 2024). Targeted probiotic intervention (e.g., emodin and pterostilbene) further expands the multi-pathway ferroptosis protection mechanism by reshaping the gut microbiota composition and inhibiting NOX2 and superoxide production (Hu et al., 2024; Zhang et al., 2026). However, there are still key bottlenecks in clinical application: the persistence and stability of microbiota transplantation, individualized response strategies based on baseline microbiota composition, and long-term safety in chemotherapy for cancer patients need to be fully characterized (Abdulaal et al., 2025; Huang et al., 2024). Therefore, large-scale prospective clinical trials are required to establish the optimal timing of FMT, donor selection criteria, and standardized protocols for probiotic strains.

Intestinal microbiota testing should focus on three levels of key indicators: (1) The abundance of short-chain fatty acid (SCFA)-producing bacteria (Faecalibacterium prausnitzii, Roseburia spp., Akkermansia muciniphila) and markers of barrier integrity (serum ZO-1, claudin, and fecal calprotectin) to evaluate GPR43/GPR41 signaling and intestinal barrier function (Huang et al., 2022; Makkieh et al., 2026); (2) The abundance of pro-inflammatory bacteria such as Proteobacteria, the ratio of lipopolysaccharide (LPS) to SCFA, and serum iron metabolism parameters (serum iron, ferritin, hepcidin), which reflect the risks of dysbiosis and iron overload (An et al., 2021; Kunika et al., 2023); (3) Plasma trimethylamine N-oxide (TMAO), circulating butyrate, and urinary phenolic metabolites, which serve as non-invasive surrogate markers of microbiota function. An increase in TMAO accompanied by a decrease in butyrate indicates susceptibility to ferroptosis (Huang et al., 2025; An et al., 2021). The dysbiosis characteristics (decreased SCFA-producing bacteria, increased pathogenic bacteria, barrier disruption, and abnormal iron metabolism) formed by the combination of these multi-dimensional markers can accurately identify high-risk patients who require early fecal microbiota transplantation or targeted probiotic intervention (Huang et al., 2024; Abdulaal et al., 2025).

## 5 Phytochemical interventions targeting ferroptosis

Research suggests that relying on a single pharmacological target is often insufficient to effectively halt the complex and progressive pathology of ferroptosis. As detailed in Tables 1–3, a diverse array of natural products—including flavonoids, polyphenols, phenylpropanoids, and terpenoids—along with traditional Chinese medicine formulas, demonstrate significant regulatory capabilities against ferroptosis. Unlike conventional free radical scavengers, these bioactive compounds operate not through a singular signaling pathway, but via synergistic multi-target and multi-pathway mechanisms. They can chelate intracellular labile iron, inhibit the enzymatic activity of the ALOX family, and epigenetically activate the Nrf2/GPX4 antioxidant axis. Collectively, these compounds offer a systematic intervention strategy, providing a fresh perspective for the prevention and treatment of ferroptosis-related diseases.

**TABLE 1:** Flavonoids targeting ferroptosis in DOX-induced cardiotoxicity.

Compound	Subclass	Experimental model	Key ferroptosis-regulatory mechanisms	Reference
Quercetin	Flavonol	*In vivo* (rats); *In vitro* (cardiomyocytes)	decreases iron accumulation (*via* BMP6/SMAD4-hepcidin, Ang II-L-type Ca channel); increases SIRT1/p53/SLC7A11 axis and GSH-dependent antioxidant capacity	Dulf et al. (2024), Guo et al. (2018), Lin X. et al. (2023), Tang et al. (2014)
Luteolin	Flavone	*In vivo* (rats)	enhances mitophagy (clears dysfunctional mitochondria)	Xu et al. (2020)
Baicalin	Flavone glycoside	*In vivo* (rats); *In vitro* (cardiomyocytes)	increases GPX4, SLC7A11, and GSH; reduces lipid peroxides	Zeng et al. (2024)
Fisetin	Flavonol	*In vivo* (mice, rats); *In vitro* (cardiomyocytes)	increases SIRT1/Nrf2 axis, GPX4, HO-1, and FTH1	Li D. et al. (2022)
Licochalcone A	Chalcone	*In vivo* (rats); *In vitro* (cardiomyocytes)	increases PI3K/AKT/MDM2/p53 axis, SLC7A11, and GPX4; decreases ACSL4, ROS, and MDA	Chen et al. (2024a), Lin J.-H. et al. (2023)
Kaempferol	Flavonol	*In vivo* (rats); *In vitro* (cell type not specified)	increases Nrf2/SLC7A11/GPX4 axis; reduces ACSL4-mediated PUFA esterification	Zhang et al. (2025)
Cyanidin chloride	Anthocyanin	*In vivo* (zebrafish)	enhances Nrf2 nuclear translocation (*via* Keap1 binding), FTH1, SLC7A11, and GPX4; decreases lipid peroxidation and free iron	Liu et al. (2023)
Hesperidin	Flavanone glycoside	*In vivo* (rats); *In vitro* (cell type not specified)	increases antioxidant enzymes (catalase, SOD); reduces non-heme iron and MDA (iron-chelating properties)	Abdel-Raheem and Abdel-Ghany (2009), Shaernejad et al. (2025)

**TABLE 2:** Polyphenols targeting ferroptosis in DOX-induced cardiotoxicity.

Compound	Subclass	Key ferroptosis-regulatory mechanisms	Experimental model	References
Resveratrol	Stilbenoid	increases SIRT1/SIRT3/Nrf2 axis and FTH1; decreases TfR1 (iron uptake) and MAPK pathways	*In Vivo* (rats/mice); *In Vitro* (cell lines)	Chen et al. (2024b) Das et al. (2015), Li T. et al. (2022), Liu et al., 2022b Wang et al. (2022), Yu et al. (2022), Zhang W. et al. (2023)
Curcumin	Diarylheptanoid	increases AMPK/Nrf2/HO-1 axis; decreases ACSL4, PTGS2, and mitochondrial ROS; also acts *via* iron chelation	*In Vivo* (rats/mice); *In Vitro* (cell lines)	Foroutan et al. (2024), Wei et al. (2022), Zhang Y. et al. (2024)
Salidroside	Phenylethanoid glycoside	increases AMPK signaling and GPX4; reduces iron accumulation; modulates fatty acid metabolism and maintains mitochondrial homeostasis	*In Vivo* (rats/mice); *In Vitro* (cell lines)	Chen et al. (2022)
6-Gingerol	Phenylalkanone	increases Nrf2/HO-1 axis and GPX4; decreases ACSL4 and iron accumulation	*In Vivo* (rats/mice); *In Vitro* (cell lines)	Wu S. et al. (2022)
Capsaicin	Vanilloid	increases PI3K-AKT axis and FPN1 (iron efflux); decreases TfR (iron import); restores intracellular iron homeostasis	*In Vivo* (rats/mice); *In Vitro* (cell lines)	Wang L. et al. (2023)

**TABLE 3:** Phenylpropanoids, terpenoids, and traditional Chinese herbal formulas targeting ferroptosis in DOX-induced cardiotoxicity.

Category/Compound/Herbal formula	Subclass/Composition	Experimental model	Key ferroptosis-regulatory mechanisms	References
Phenylpropanoids and terpenoids				
Cinnamaldehyde	Phenylpropanoid aldehyde	*In vivo* (rats); *In vitro* (cardiomyocytes)	increases Nrf2/HO-1 signaling and Nrf2 nuclear translocation; decreases ROS and iron accumulation	Mao et al. (2023)
Astragaloside IV	Triterpene saponin	*In vivo* (rats); *In vitro* (cardiomyocytes)	increases Nrf2/GPX4 antioxidant axis; decreases NOX2/NOX4 and CD36-mediated lipid uptake	Li X. et al. (2023), Lin et al. (2019), Luo et al. (2021)
Tanshinone IIA	Phenanthrenequinone diterpene	*In vivo* (rats); *In vitro* (HCAEC)	increases Nrf2/SLC7A11/GPX4 cascade, GSH, and FTH1; reduces lipid ROS production	He et al. (2021)
Traditional Chinese herbal formulas				
Salvia miltiorrhiza Injection	Aqueous extract of Salvia miltiorrhiza Bunge	*In vivo* (mice)	decreases myocardial iron deposition, lipid peroxidation, and injury markers (MDA, CK-MB, LDH); increases antioxidant enzymes (SOD, GPX); restores ferroptosis-resistant state	Zhang et al. (2013), Zhang et al. (2015)
QiShenYiQi dripping pill	Astragalus membranaceus, Salvia miltiorrhiza, Panax notoginseng, Dalbergia odorifera	*In vivo* (rats); *In vitro* (cell type not specified)	increases GPX4 and SLC7A11; decreases ACSL4 and PTGS2; restores mitochondrial dynamics and biogenesis	Wu et al. (2023)
Ling-Gui-Zhu-Gan decoction	Poria cocos, Cinnamomum cassia, Atractylodes macrocephala, Glycyrrhiza uralensis	*In vivo* (rats); *In vitro* (cell type not specified)	increases Nrf2 signaling, GPX4, and FPN1 (iron efflux); decreases PTGS2-driven lipid peroxidation	Yang Y. et al. (2024)

Sufficient evidence has been accumulated regarding the clinical application of phytochemicals in patients with doxorubicin-induced cardiotoxicity. A meta-analysis incorporating multiple clinical studies indicates that the application of traditional Chinese medicine as adjuvant therapy for patients receiving anthracycline chemotherapy can significantly reduce the incidence of cardiotoxicity-related adverse events, and its cardioprotective effect is well-defined (Hao et al., 2022). In traditional Chinese medicine, Salvia miltiorrhiza is widely used to prevent doxorubicin-induced cardiotoxicity. Clinical studies have confirmed that Salvia miltiorrhiza preparations can improve the left ventricular ejection fraction and myocardial injury markers in patients (Yue et al., 2025). As a classic cardioprotective prescription, Shengmai San has been shown to significantly reduce the incidence of cardiotoxicity in anthracycline chemotherapy patients compared with the control group (Zhang et al., 2022). Shenqi injection, a commonly used adjuvant treatment regimen, has also been verified in clinical practice, especially in significantly reducing the elevation of cardiac injury markers in chemotherapy patients (Yang L. et al., 2024; Meng et al., 2022). In terms of monomeric natural products, black seed oil has demonstrated its cardioprotective effect in the clinical application for children with acute lymphoblastic leukemia, and the markers of doxorubicin-induced cardiotoxicity in patients receiving this treatment were significantly reduced (Hagag et al., 2020). The application of Ginkgo biloba extract in patients treated with doxorubicin in clinical practice shows that it can effectively reduce the elevation of myocardial endothelin-1 and abnormal nitric oxide levels, and improve doxorubicin-induced acute cardiotoxicity (El-Boghdady, 2013). Additionally, the results of a randomized controlled trial on early breast cancer patients receiving anthracycline chemotherapy while using Platycodon grandiflorum preparations show that this plant preparation can reduce the incidence and severity of cardiotoxicity in patients (Hao et al., 2017). Pomegranate extract (Fruitflow®) has also been confirmed in clinical studies to have the effect of protecting cardiomyocytes from doxorubicin-induced damage (Das et al., 2024). These clinical evidences suggest that phytochemicals can effectively reduce the risk of doxorubicin-induced cardiotoxicity through multi-targeted intervention in key pathological mechanisms such as mitochondrial function (Sun et al., 2022), oxidative stress, and inflammatory response while maintaining anti-tumor efficacy, and thus have substantial clinical application value.

## 6 Balancing cardiac safety and antitumor efficacy during DOX-based chemotherapy

There is a core contradiction in ferroptosis: it is not only the key mechanism for doxorubicin to exert its anti - tumor effect but also mediates its cardiotoxicity. Tumor cells and cardiomyocytes share a set of core ferroptosis regulatory networks, among which the Nrf2/GPX4/SLC7A11 axis, iron homeostasis maintenance, and lipid peroxidation processes are the core components. This commonality makes it difficult to balance cardioprotection and anti - tumor effects simultaneously through systemic regulation of ferroptosis. The key to solving this problem lies in constructing a selective intervention strategy to achieve precise targeted regulation of the ferroptosis pathway in different tissues.

### 6.1 Time-decoupling strategy based on IFN-γ regulation

IFN-γ signaling exhibits a high degree of environmental dependence during doxorubicin (DOX) treatment, presenting a “double-edged sword” effect: it is crucial for the anti-tumor immune response, yet it significantly exacerbates the sensitivity of cardiomyocytes to ferroptosis and collateral damage. The core theoretical basis for targeting IFN-γ lies in its pleiotropic mechanisms within the cardiac microenvironment. Research indicates that IFN-γ not only directly mediates immune cardiac injury through the macrophage-driven IFN-γ-STAT1 signaling axis, a pathway known to transcriptionally suppress the ferroptosis defense gene SLC7A11 (Zhang et al., 2026), but also triggers cardiac conduction abnormalities via the IL-18-IFN-γ-Cx43 cascade (Li et al., 2025). More importantly, Ji et al. (2025) confirmed that IFN-γ can reprogram cardiac microvascular endothelial cells, actively mediating and increasing the transport and accumulation of DOX in cardiac tissue. Clinical historical data also support this mechanism; for example, the treatment of lung cancer patients with recombinant IFN-γ directly led to cardiotoxicity (Mattson et al., 1991).

To address this contradiction, a “temporal decoupling” strategy exploits the pharmacokinetic and pharmacodynamic time windows between tumor immune activation and the accumulation of myocardial oxidative stress. In preclinical models, Ma et al. (2020) demonstrated that transient blockade of IFN-γ with the R46A2 antibody can effectively alleviate DOX-induced myocardial injury without compromising its anti-tumor efficacy. The precision of this temporal intervention lies in the fact that early IFN-γ signaling is sufficient to initiate tumor immunity, while subsequent blockade can prevent the continuous, IFN-γ-driven endothelial transport of DOX (Ji et al., 2025) and the subsequent STAT1-mediated cardiac ferroptosis.

However, the clinical translation of this strategy faces multiple challenges and potential risks. Firstly, precise timing control is crucial, and there is currently no mature method for clinically monitoring IFN-γ levels dynamically and determining the optimal blockade time window. Secondly, systemic IFN-γ blockade may lead to broad-spectrum suppression of the immune system, increasing the risk of infection or affecting the efficacy of other concomitant immunotherapies. Thirdly, patient heterogeneity (such as differences in tumor type, immune microenvironment, and individual myocardial responses to DOX) may make it difficult to apply a unified temporal decoupling strategy to all patients. Additionally, the lack of reliable biomarkers to predict the risk of IFN-γ-mediated cardiac injury or guide the timing of intervention is also a major obstacle to its clinical application. Successful translation requires balancing the needs of individualized treatment with the complexity of systemic immune regulation.

### 6.2 Cardiac-targeted drug delivery: the OGF/OGFR paradigm

Organ-selective delivery strategies can achieve specific regulation of the same ferroptosis pathway in different tissues, fundamentally reducing off-target effects (Allijn et al., 2017; Ferreira et al., 2017). The OGF-OGFR axis plays a crucial role in chemotherapy-induced myocardial injury (Liu X. et al., 2022). After DOX exposure, the accumulation of OGF and the upregulation of OGFR expression in cardiac tissue lead to the imbalance of redox homeostasis in cardiomyocytes and the impairment of ferroptosis defense, thereby exacerbating DOX-induced oxidative damage (Liu C. et al., 2022). However, since the OGF-OGFR axis is a homeostatic regulator that restricts cell proliferation (McLaughlin and Zagon, 2012; Zagon et al., 2002), systemic blockade of OGFR significantly reduces the anti-tumor effect of DOX. Chen et al. (2024a) achieved both myocardial protection and anti-tumor efficacy in a mouse model using heart-targeted naltrexone nanoparticles, for the first time validating the feasibility of this organ-separated pharmacological strategy.

Despite its broad prospects, this method still faces numerous challenges and potential risks in clinical translation: 1) Insufficient validation of tissue selectivity: Quantitative biodistribution studies are required to strictly compare the expression of OGFR and the accumulation of nanoparticles in the heart and tumors to ensure heart-specific delivery and avoid new toxicities caused by accumulation in non-target organs; 2) Tumor heterogeneity: Different cancer types may respond differently to the DOX-OGFR combination therapy, affecting the universality of treatment; 3) Transformation optimization: Refinement of heart-targeted ligands and nanoparticle formulations is crucial for clinical deployment. Technical challenges such as the immunogenicity, *in vivo* stability, and drug loading of nanoparticles need to be overcome.

### 6.3 Cross-regulation between ferroptosis and related cell death pathways

The crosstalk among ferroptosis, cuproptosis, and mitochondrial apoptosis provides new therapeutic directions for doxorubicin (DOX)-induced cardiotoxicity. Given that single-pathway blockade often triggers compensatory cell death (i.e., “death escape”), simultaneously targeting these interrelated modes has unique advantages in preventing “death escape” (Yang Y. et al., 2024). Essentially, these cell death pathways converge on common hubs, mainly including mitochondrial dysfunction and reactive oxygen species (ROS) burst (Tai et al., 2023; He et al., 2025). Therefore, therapeutic strategies that synergistically inhibit multiple pathways demonstrate excellent cardioprotective effects. For example, BRD4770 potently protects cardiomyocytes by simultaneously blocking apoptosis and ferroptosis (Shao X. et al., 2025); the SIRT7 activator aprocitentan can simultaneously inhibit cuproptosis, oxidative stress, and mitochondrial dysfunction (Chen et al., 2025); and trifolirhizin-mediated FDX1 inhibition restores mitochondrial bioenergetics by inhibiting cuproptosis without compromising anti-tumor efficacy (Wei et al., 2025). By systematically targeting these shared mitochondrial stress nodes, it is promising to achieve the dual goals of preserving anti-tumor activity while protecting the heart.

However, introducing this multi-pathway cross-regulation strategy into clinical practice faces significant challenges and potential risks: 1) Drug development complexity: It is extremely difficult to design drugs that can precisely target multiple cell death pathways simultaneously, ensure selective inhibition of cell death in the heart, and maintain killing effects in tumors. 2) Off-target effects of broad-spectrum inhibition: Cell death pathways are fundamental components of cell physiology. Broad-spectrum inhibition of these pathways may lead to unpredictable systemic side effects in healthy tissues, such as affecting immune cell function or normal cell turnover. 3) Balance between dosage and toxicity: Maintaining anti-tumor activity while protecting the heart requires a delicate dosage balance, as the sensitivity or regulatory mechanisms of these pathways may vary in different tissues. 4) Drug interactions: If combination therapy is required to target multiple pathways, complex drug interactions will be an important clinical consideration. 5) Lack of reliable biomarkers: Currently, there is a lack of effective biomarkers to accurately monitor the effects and potential side effects of multi-death pathway inhibition. 6) Unknown long-term safety: Long-term inhibition of these key cell death pathways may have unknown long-term effects on patients.

### 6.4 Tissue-specific ferroptosis regulation

The core idea of this strategy is to promote ferroptosis (or inhibit its ferroptosis defense mechanism) in tumor tissue and selectively inhibit ferroptosis in myocardial tissue, so as to achieve a balance between efficacy and safety. Relevant research in the field of radiotherapy provides strong support for this idea: Lei et al. (2020) found that ionizing radiation can induce ferroptosis in radiotherapy - sensitive tumors, while drug - resistant tumors can escape ferroptosis by up - regulating the expression of GPX4 and SLC7A11. Inactivating these two genes can restore the radiotherapy sensitivity of tumors. Combining with existing delivery technologies, this strategy can be achieved in two ways: one is to use hypoxia - activated prodrugs, and the other is to use tumor - targeted peptide - modified nanocarriers to accurately deliver ferroptosis sensitizers (such as SLC7A11 inhibitors and GPX4 inhibitors) to tumor tissue.

## 7 Conclusion and future perspectives

### 7.1 Existing bottlenecks in myocardial protection research based on ferroptosis

Translating the research findings of ferroptosis into clinical strategies for alleviating DIC still faces numerous core challenges. Apart from the fact that the regulatory mechanism of ferroptosis has not been fully elucidated, the biggest bottleneck lies in the fact that if myocardial protection is to be achieved by inhibiting ferroptosis in cardiomyocytes, the anti - tumor efficacy of doxorubicin will inevitably be weakened (Dai et al., 2024; Sies et al., 2024; Zheng and Conrad, 2025). This inherent contradiction cannot be fundamentally resolved even by optimizing the dosage and adjusting the administration timing.

The complexity of ferroptosis regulation further magnifies this research dilemma. It is not a single linear pathway but a multi - dimensional network intertwined with iron homeostasis, phospholipid remodeling, the glutathione/glutathione peroxidase 4 (GSH/GPX4) - dependent antioxidant system, mitochondrial integrity, and metabolic signals. All these links are dynamically regulated by the cellular microenvironment (Dai et al., 2024). As a result, current target screening mostly relies on empirical exploration, and there is a lack of unified standards for mechanism interpretation among different experimental models, ultimately limiting the reproducibility of research results.

### 7.2 Underestimated complexity: autophagy, ferroptosis plasticity, and limitations of biomarkers

In most current studies, the complexity of the autophagy mechanism is often overlooked. Autophagy is not simply a cell - protective mechanism, and its effect has a significant duality. On the one hand, it can mobilize the labile iron pool and oxidizable lipids in cells to accelerate the ferroptosis process; on the other hand, it can also initiate the cell’s own defense response to resist ferroptosis - related damage. This bidirectional regulatory effect varies with cell type, nutritional supply status, and pathway activation background. However, current research on DIC has not delved deeply enough into these regulatory factors.

The metabolic process of ferroptosis also has strong plasticity and can dynamically adapt according to the nutritional conditions of cells (such as selenium availability and iron acquisition efficiency) and related metabolic pathways (such as cysteine/methionine metabolism, mevalonate synthesis, and phospholipid remodeling), directly reducing the predictability of ferroptosis - targeted therapy (Zeng et al., 2023).

Currently, in clinical practice, the traditional biomarkers used for assessing cardiac injury are primarily high-sensitivity cardiac troponin I (hs-cTnI) and B-type natriuretic peptide (BNP) (Bhagat and Kleinerman, 2020). Among them, hs-cTnI can sensitively diagnose acute myocardial injury and predict the early decline of left ventricular ejection fraction (LVEF), but it is unable to evaluate long-term myocardial remodeling and late cardiac toxicity (Hsu et al., 2026). Myeloperoxidase (MPO), an oxidative stress-related pro-inflammatory enzyme secreted by neutrophils, can also serve as an auxiliary biomarker for assessment (Bhagat and Kleinerman, 2020).

Compared with traditional biomarkers, a variety of emerging biomarkers demonstrate higher application value in the early warning and assessment of cardiac toxicity and can be mainly classified into four categories: 1) Circulating microRNAs: including miR-34a-5p, miR-122-5p, miR-155-5p, miR-1a-3p, etc. The expression level of miR-34a-5p increases in a dose-dependent manner with the increase of drug dosage and can be regulated by cardioprotective drugs, which can effectively identify early and late delayed cardiac toxicity (Desai et al., 2022; Zhang R. et al., 2023). miR-122-5p can be used to evaluate the severity of cardiac toxicity and predict clinical adverse reactions (Zhang H. et al., 2024). 2) Blood metabolites: Metabolites such as amino acids, acylcarnitines, and phospholipids are significantly correlated with cardiac function indicators. They can non-invasively reflect myocardial metabolic injury and be used to judge the prognosis of cardiac toxicity (Thonusin et al., 2023; Thonusin et al., 2024). DNA methylation biomarkers: The cardiac-specific methylation biomarker mPIH1D1 is highly correlated with ventricular dilation (AUC = 0.95, sensitivity 100%, specificity 90.7%). It can identify myocardial structural damage earlier than troponin and predict long-term ventricular remodeling (Hsu et al., 2026). 3) Inflammation-related biomarker Limd1 can regulate the infiltration state of immune cells and has good diagnostic efficacy (AUC = 0.847), thus revealing the intrinsic relationship between immune infiltration and cardiac toxicity (Zhang W. et al., 2023). 4) Cytokine GDF15: This factor belongs to stress-responsive cytokines, which are upregulated after the activation of the integrated stress response (ISR) and participate in the regulation of proteostasis stress. It is a potential specific biomarker for mitochondrial dysfunction (Wang et al., 2026).

Integrating multi-level biomarkers, especially combining cardiac-specific biomarkers such as mPIH1D1 with hs-cTnI, can provide support for the early screening and dynamic monitoring of clinical cardiac toxicity and contribute to the development of individualized cardioprotective strategies and risk stratification plans based on the patient’s HER2 expression status and chemotherapy regimen.

### 7.3 Obstacles to clinical translation

Although a variety of measures have been established for the clinical prevention and treatment of doxorubicin cardiotoxicity, including dose limitation (≤400 mg/m^2^), optimization of drug administration methods, intervention with neurohormonal inhibitors, and the use of dexrazoxane (Gao et al., 2023; Gulati et al., 2016), significant issues still exist in global clinical implementation. A survey in the Netherlands revealed substantial differences among different medical institutions in the dosage standards and equivalent factors of anthracycline chemotherapeutic agents, with most institutions failing to routinely screen for left ventricular ejection fraction (Ditta et al., 2026). In terms of evidence quality, the quality of existing international guidelines is generally low, with high-quality evidence comprising only 11% of current recommendations (Moustafa et al., 2023). These limitations stem from: (1) the lack of consistent standards in clinical implementation; (2) insufficient stratification of individual patient risks; and (3) the limited protective effect of single-target intervention.

The multi-target intervention strategy based on ferroptosis regulation mechanisms provides a novel approach to overcome these bottlenecks. First, optimize drug delivery through novel nanodelivery systems. Although natural compounds (such as flavonoids and plant polyphenols) have demonstrated significant myocardial repair capabilities in both *in vitro* and *in vivo* experiments (Nsairat et al., 2025; Viswanatha Swamy et al., 2011; Shang et al., 2025), they are severely limited by extremely low bioavailability. To address this critical limitation, emerging nanodelivery systems represent a paradigm shift in cardioprotection strategies. Lignin nanoliposomes provide a biocompatible carrier system derived from renewable resources, enabling efficient encapsulation and targeted myocardial delivery of cardioprotective agents (Shi et al., 2025). Plant-derived extracellular vesicles offer intrinsic targeting capabilities and can modulate cardiomyocyte ferroptosis through natural bioactive components (Liu et al., 2026). Chitosan-modified exosomes utilize their cationic surface properties to enhance cellular uptake while maintaining compatibility with doxorubicin’s anticancer mechanisms (Jiang et al., 2026; Wen Z. et al., 2024). These nanocarriers not only enhance drug bioavailability 5- to 10-fold compared to conventional administration but also enable site-specific accumulation in injured myocardial tissue through passive or active targeting mechanisms, thereby facilitating ferroptosis inhibition via GPX4 stabilization and iron chelation. Second, establish a multi-mechanism combined intervention plan centered on ferroptosis. SGLT2 inhibitors and metformin can regulate multiple metabolic and inflammatory pathways, with myocardial protective effects superior to those of traditional neurohormonal blockers (Oh et al., 2019; Liu et al., 2018). Notably, the NCT06888505 clinical trial is currently verifying the cardioprotective effects of dapagliflozin in patients receiving anthracycline chemotherapy. The miRNA-targeting antagonist AM106 can inhibit pathological myocardial remodeling, with evidence demonstrating simultaneous achievement of both anti-tumor and cardioprotective effects (Asensio-Lopez et al., 2025). By simultaneously implementing lipid peroxidation inhibition, enhancing GPX4 activity and GSH levels, regulating iron homeostasis, and maintaining protein quality control, synergistic myocardial protection can be achieved within a unified pathophysiological framework. This comprehensive approach circumvents the safety concerns associated with high-concentration vitamin C, which may compromise chemotherapy efficacy and promote tumor proliferation (Nsairat et al., 2025; Heaney et al., 2008; Viswanatha Swamy et al., 2011). Third, refine precise risk stratification and dynamic monitoring systems. Multidisciplinary teams can significantly improve long-term outcomes in high-risk patients through stratified management based on baseline cardiovascular risk profiles, supported by multi-target pharmacological regimens, dynamic biomarker monitoring, and adjunctive non-pharmacological interventions (Cardinale et al., 2015). Although mitochondrial-targeted interventions (such as mitochondrial transplantation, modulation of mitochondrial fission, and autophagy enhancement) can effectively restore damaged myocardial function (Wu S. et al., 2022), critical challenges remain regarding immune tolerance and *in vivo* delivery technology. Finally, standardize the accumulation of clinical evidence and guideline development. Through rigorously designed clinical trials, the effectiveness and safety of ferroptosis-based strategies must be systematically compared with traditional approaches, thereby establishing the high-quality evidence foundation necessary for formulating unified international guidelines and addressing the current deficiencies in guideline quality and evidentiary support (Heck et al., 2021; Moustafa et al., 2023).

This systematic translational strategy from mechanistic discovery to clinical implementation will fundamentally transform doxorubicin cardiotoxicity prevention from empirical, single-target interventions into a comprehensive, mechanism-oriented, precisely individualized, and multidisciplinary approach, ultimately providing standardized and scalable solutions for clinical practice.

## Glossary

4-HNE: 4-hydroxynonenal
ACSL4: Acyl-CoA Synthetase Long-Chain Family Member 4
AKT: Protein Kinase B
ALKBH5: AlkB Homolog 5
ALOX: Lipoxygenase
AMPK: AMP-Activated Protein Kinase
Ang II: Angiotensin II
ASM: Acid Sphingomyelinase
BACH1: BTB Domain and CNC Homolog 1
BMP6: Bone Morphogenetic Protein 6
CD36: Cluster of Differentiation 36
CMA: Chaperone-Mediated Autophagy
CRISPR: Clustered Regularly Interspaced Short Palindromic Repeats
DIC: Doxorubicin-Induced Cardiotoxicity
DOX: Doxorubicin
Drp1: Dynamin-Related Protein 1
EHA: European Hematology Association
ER-MITO: Endoplasmic Reticulum-Mitochondria
ESC: European Society of Cardiology
ESTRO: European Society for Therapeutic Radiology and Oncology
FDA: Food and Drug Administration
FDX: Ferredoxin
FDX1: Ferredoxin 1
Fer-1: Ferrostatin-1
FMT: Fecal Microbiota Transplantation
FPN1: Ferroportin 1
FTH1: Ferritin Heavy Chain 1
FTL: Ferritin Light Chain
FUNDC1: FUN14 Domain Containing 1
FXR: Farnesoid X Receptor
GPX4: Glutathione Peroxidase 4
GPR: G-protein-coupled Receptor
GSH: Reduced Glutathione
HDAC: Histone Deacetylase
HO-1: Heme Oxygenase-1
IC-OS: International Cardio-Oncology Society
IFN-γ: Interferon-gamma
KAT5: Lysine Acetyltransferase 5
KEAP1: Kelch-Like ECH-Associated Protein 1
LC3: Microtubule-Associated Protein 1 Light Chain 3
Lip-1: Liproxstatin-1
LOOH: Lipid Hydroperoxides
LPS: Lipopolysaccharide
**m** ^**6**^ **A**: N6-methyladenosine
MAPK: Mitogen-Activated Protein Kinase
MDA: Malondialdehyde
MDM2: Mouse Double Minute 2
MERC: Mitochondria–ER Contact Sites
MG53: Muscle RING Finger Proteins 53
mtROS: Mitochondrial Reactive Oxygen Species
NADPH: Nicotinamide Adenine Dinucleotide Phosphate
NCOA4: Nuclear Receptor Coactivator 4
NOX: NADPH Oxidase
NRF2: Nuclear Factor Erythroid 2-Related Factor 2
OGF: Opioid Growth Factor
OGFR: Opioid Growth Factor Receptor
PI3K: Phosphatidylinositol 3-Kinase
PKA: Protein Kinase A
PLOOH: Phospholipid Hydroperoxides
PTGS2: Prostaglandin-Endoperoxide Synthase 2
PUFA: Polyunsaturated Fatty Acids
ROS: Reactive Oxygen Species
SCFA: Short-Chain Fatty Acids
SIRT1: Sirtuin 1
SIRT3: Sirtuin 3
SIRT6: Sirtuin 6
SLC7A11: Solute Carrier Family 7 Member 11
SMAD4: SMAD Family Member 4
SOD: Superoxide Dismutase
SOD2: Superoxide Dismutase 2
TfR1: Transferrin Receptor 1
TMAO: Trimethylamine N-Oxide
TNF-α: Tumor Necrosis Factor-α
Top2β: Topoisomerase IIβ
Treg: Regulatory T Cells
UPS: Ubiquitin-Proteasome System
USP10: Ubiquitin Specific Peptidase 10
USP51: Ubiquitin Specific Peptidase 51
VDAC1: Voltage-Dependent Anion Channel 1
YAP: Yes-Associated Protein
YTHDF2: YTH Domain Family Protein 2
